# Genome-Wide Association Study and RNA-Seq Elucidate the Genetic Mechanisms Behind Aphid (*Rhopalosiphum maidis* F.) Resistance in Maize

**DOI:** 10.3390/plants14111614

**Published:** 2025-05-25

**Authors:** Doudou Sun, Yijun Wei, Chunyan Han, Xiaopeng Li, Zhen Zhang, Shiwei Wang, Zijian Zhou, Jingyang Gao, Jiafa Chen, Jianyu Wu

**Affiliations:** 1 College of Life Sciences, Henan Agricultural University, Zhengzhou 450046, China; sundd@henau.edu.cn (D.S.); zhouzijian19900601@henau.edu.cn (Z.Z.);; 2 Postdoctoral Station of Crop Science, Henan Agricultural University, Zhengzhou 450046, China; 3 State Key Laboratory of High-Efficiency Production of Wheat-Maize Double Cropping, Henan Agricultural University, Zhengzhou 450046, China

**Keywords:** aphid, maize, association analysis, RNA-seq, molecular breeding

## Abstract

Maize is a crucial food crop and industrial raw material, significantly contributing to national food security. Aphids are one of the most prevalent and destructive pests in maize production, necessitating the exploration of pest-resistant germplasm and the development of resistant varieties as the most fundamental and effective strategy for mitigating aphid-induced damage. This study established an aphid resistance evaluation system and identified 17 elite resistant inbred lines through multi-year screening. A genome-wide association study (GWAS) revealed 22 significant single-nucleotide polymorphisms (SNPs) associated with aphid resistance, including genes involved in benzoxazinoid (Bx) biosynthesis (such as *Bx2*), insect resistance-related transcription factors (such as *WRKY23*), plant lectins, and other resistance pathways. RNA-seq analysis of the samples before and after aphid infestation detected 1037 differentially expressed genes (DEGs) in response to aphid infestation, with KEGG enrichment highlighting benzoxazinoid biosynthesis and starch/sucrose metabolism as primary response pathways. Integrating GWAS and RNA-seq results revealed the presence of several benzoxazinoid synthesis-related genes on the short arm of chromosome 4 (Chr4S). FMqRrm1, a Kompetitive Allele-Specific PCR (KASP) marker, was derived from the Chr4S region. We subsequently utilized this marker for marker-assisted selection (MAS) to introgress the Chr4S region from the aphid-resistant inbred line into two aphid-susceptible inbred lines. The results demonstrated that the Chr4S favorable allele significantly reduced aphid occurrence by 1.5 to 2.1 grades. This study provides a critical theoretical foundation and practical guidance for understanding the molecular mechanism of aphid resistance in maize and molecular breeding for aphid resistance.

## 1. Introduction

Maize (*Zea mays* L.) is an essential grain crop and industrial raw resource with significant implications for national food security. Maize aphid (*Rhopalosiphum maidis* F.) is a common and destructive corn pest worldwide that reduces corn productivity by 10–20% in a typical year. The primary cause of damage is the large amounts of honeydew it excretes on the plant surface while feeding, which has a detrimental effect on the leaves’ ability to photosynthesize. Its stinging and piercing-sucking mouthparts also cause wilting and yellowing of the leaves, which consequently leads to severe stunting of the maize plant [1]. Aphids can also transmit several maize viruses, including the Sugarcane Mosaic Virus (SCMV) and Maize Rough Dwarf Virus (MRDV). Studies show that the indirect yield loss caused by aphids through virus transmission is significantly higher than the direct yield loss [2,3].

The currently prevalent chemical methods for aphid control in agricultural production may lead to severe environmental pollution and pose threats to human health. Although new aphid control methods are emerging [4], breeding and promoting aphid-resistant varieties remains the most economical and environmentally benign strategy for aphid control [5]. Previous research has demonstrated that maize varieties and germplasm resources varied significantly in their resistance to aphids. For example, most of the germplasm in SS (Stiff-Stalk) heterosis groups represented by PH6WC (the female parent of hybrid Xianyu335) is particularly susceptible to aphids. Furthermore, China’s recently published insect-resistant transgenic safety certificates do not include any aphid-resistant transgenic events [6]. Consequently, the most promising aphid resistance breeding strategies involve mining resistant germplasm resources, identifying resistant genes/QTL (quantitative trait loci), and breeding resistant varieties.

Previous studies have demonstrated that the endogenous levels of abscisic acid (ABA) and jasmonic acid (JA) are significantly correlated with aphid resistance [7,8]. In addition, leaf surface wax composition and soluble sugar content influence aphid feeding behavior and reproductive capacity [9]. Furthermore, the accumulation of secondary metabolites, including diterpenoids and DIMBOA (2,4-dihydroxy-7-methoxy-1,4-benzoxazin-3-one), has been shown to play a crucial role in plant defense against aphid infestation [10]. In particular, the concentration of DIMBOA is strongly related to aphid resistance, and DIMBOA-Glc (DIMBOA-glucoside), the most common storage precursor of DIMBOA in cells, also plays a significant role in the mechanism of maize aphid resistance [1,11].

Identifying resistance-related genes/QTL based on linkage and association analysis is a common genetic approach [12,13,14]. DIMBOA has been shown to confer resistance to aphids, with several QTL identified in DIMBOA synthesis across a cohort of genetically diverse maize inbreds, using eight RIL (replicated inbred line) families from the nested association mapping population [15]. Aphid feeding reduced the accumulation of 5-hydroxynorvaline (5-HDA) in maize leaves. Subsequent analysis identified two QTLs on chromosomes 5 and 7 associated with 5-HDA accumulation [16]. The RIL population created with B73 and CML322 as parents also employed DIMBOA and HDIMBOA as phenotypes to pinpoint the aphid resistance site to region 1.04 [17]. Another study employed B73 and Mo17 as parents to construct linkage populations, and the number of aphids and DIMBOA content served as indicators for genetic studies, with loci on chromosomes 4 and 6 mapped, respectively [18]. Recently, the DIMBOA contents of 310 maize inbred lines were calculated and used for marker-trait association analysis, which detected 49 candidate genes within 19 hot loci including 12 controlling DIMBOA biosynthesis [19]. Collecting and analyzing honeydew produced after aphids infested tobacco revealed that *NtWRKY28* positively regulates the expression of multiple genes associated with flavonoid and lignin biosynthesis and increases tobacco’s resistance to aphids [20].

Genome-wide association study (GWAS) has demonstrated significant potential for detecting causal genes/QTL with high resolution in diverse germplasm [21]. In recent decades, GWAS has been used successfully in maize to identify several causal genomic loci associated with a wide range of traits [22,23,24,25,26,27]. RNA sequencing (RNA-seq) is a high-throughput sequencing technology based on next-generation sequencing (NGS) that first appeared in 2008 [28]. It allows for the qualitative and quantitative study of all transcripts in a cell, tissue, or organism [29]. This approach is often used to detect alterations in gene expression under various conditions, including health and disease states, medication therapy, and developmental stages [30,31,32,33]. This technique makes it easier to identify differentially expressed genes (DEGs), which helps us understand the molecular processes underlying these conditions [29].

We carried out a GWAS in a diverse maize population to identify the candidate genes associated with aphid resistance. We also performed transcriptomic analyses at 0, 6, 12, and 24 h post-infestation. The integration of GWAS and RNA-seq methodologies helped address the following three objectives: (i) exploring the genetics underlying aphid resistance, (ii) identifying genes or pathways that respond to aphid infestation, and (iii) identifying candidate resistance chromosome regions by integrating GWAS and transcriptome data. This study will enhance our understanding of the molecular mechanisms underlying aphid resistance while also providing resistance loci for breeding.

## 2. Materials and Methods

### 2.1. Germplasm Materials and Experimental Design

The GWAS population comprised 278 high-quality inbred lines with domestic and international origins [24]. Including heterotic groups of Stiff Stalk(SS), Tangsipingtou, Lancaster, and tropical lines from the International Maize and Wheat Improvement Center (CIMMYT) ensured broad genetic diversity. The GWAS population was evaluated at the Hainan Experimental Station (Hainan, China; 18°26′ N, 108°58′ E) of Henan Agricultural University during the winter seasons of 2017 (E1), 2018 (E2), and 2019 (E3). The experiment was set up in a randomized full-block design with two replications, with field planting rows 4 m long, 0.6 m apart, and 0.22 m between plants. Except for the absence of aphid control measures, all other field management practices adhered to standard agricultural protocols.

### 2.2. Phenotypic Data Collection

Years of observation have shown that the annual aphid (*Rhopalosiphum maidis* F.) occurrence in Hainan Province, China, is relatively stable, making it an ideal location for natural aphid resistance identification. When the natural outbreak of aphids occurred in the fields of Hainan, significant variations in resistance were observed among different maize inbred lines while maintaining stable intra-variety performance. The experiment strictly controlled insecticide application to ensure natural aphid population development. The survey was conducted at 20 days after pollination (the period of peak aphid infestation and when the leaves are not yet dry). In this stage, systematic recordings of aphid cover area were made on each plant’s tassel and three subtending leaves.

**Figure 1:**
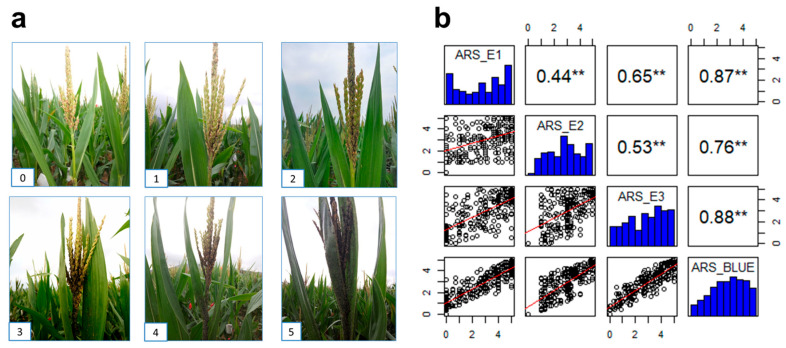
The phenotype for aphid resistance in the GWAS population. (**a**) The typical aphid resistance score (ARS) grades 0 to 5. (**b**) Distribution and correlation of aphid resistance score across environments. ** *p* < 0.01.

Then, the aphid resistance phenotypes were graded from 0 to 5 based on the degree of damage to the male spike and lower leaves. Single-plant investigations were conducted in each row, with grade 0 indicating no aphid infection, implying high resistance, and grade 5 indicating that the entire male spike and lower leaves were infected by aphids, implying high susceptibility. Table 1 details the classification criteria for varying disease severity levels, while Figure 1a depicts the distinct phenotypic expressions schematically. The disease evaluation for each material was calculated by averaging the disease scores of all the individual plants in the plot. Consequently, a scientific resistance evaluation system was established. The resistance grade is categorized as follows: 0 < resistance grade < 1 as highly resistant (HR), 1 ≤ grade < 2 as resistant (R), 2 ≤ grade < 3 as moderately resistant (MR), 3 ≤ grade < 4 as susceptible (S), and 4 ≤ grade < 5 as highly susceptible (HS).

### 2.3. Statistical Analyses for Phenotypic Data

Descriptive statistical analyses were performed using Microsoft Excel 2024, which included the generation of histogram plots, the determination of mean and range values, and the evaluation of Pearson correlation coefficients across phenotypic traits. The Multiple Environment Traits Analysis Package (META-R v6.0.4, http://hdl.handle.net/11529/10201, accessed on 12 April 2025) was used for statistical studies such as Analysis of Variance (ANOVA) and Best Linear Unbiased Estimates (BLUEs). The BLUEs were estimated using a mixed linear model, with genetic lines as fixed effects and environment, line × environment interaction, replication within the environment, and block within replication as random effects.

### 2.4. Genome-Wide Association Study

The CTAB method was used to extract DNA from young maize leaves. Their quality, purity, and concentration were determined using gel electrophoresis and a spectrophotometer (NanoDrop 8000, Thermo Fisher Scientific, Waltham, MA, USA). Inbred lines exhibiting high-quality DNA were genotyped using 5× genome resequencing [34]. After filtering out SNPs with missing ratios below 0.25 and minimum allele frequencies above 0.05, 229,379 high-quality SNP markers were retained for further analysis. The marker allele frequency, heterozygosity, and missing data were computed with TASSEL v5.0 software (https://www.maizegenetics.net/tassel, accessed on 9 March 2024) [35]. The TASSEL v5.0 software was also used to create a mixed linear model (MLM) that included BLUEs, markers, a kinship matrix (K), and principal component analysis (PCA). The MaizeGDB genome database (http://www.maizegdb.org/, accessed on 5 April 2025) was used to identify candidate genes containing or close to significant SNPs. The phytozome database (http://phytozome.jgi.doe.gov/pz/portal.html/, accessed on 5 April 2025) was used to identify key biochemical pathways and annotate the functions of candidate genes [36].

### 2.5. Total RNA Extraction and Sequencing

The seeds of the maize inbred line BT-1 were sown in dish cavities filled with a 3:1 mixture of nutrient-rich soil and vermiculite and grown under a 16:8 light-to-dark cycle in greenhouses. At the four-leaf stage, 50 adult aphids were artificially inoculated on the basal part of the seedling’s intermediate leaf with a moist brush, and the inoculation duration was diligently recorded. A 100-mesh insect-proof net was subsequently employed to isolate the seedlings and prevent aphid escape. Samples were collected at 0, 6, 12, and 24 h following aphid infestation and immediately stored in liquid nitrogen.

Total RNA was extracted from frozen leaves using the TransZol Plant kit (TransGen Biotech, Beijing, China). A microgram of RNA from each sample was used for complementary DNA (cDNA) synthesis using the HiScript III 1st Strand cDNA Synthesis Kit with a gDNA wiper (#R312-02; Vazyme, Nanjing, China). GENE READ Company (http://www.genereadtech.com/, accessed on 5 April 2025) performed RNA sequencing, and the sequence data obtained were matched to the maize reference genome of inbred line B73 (*Zea mays* B73_RefGen_v4, https://ensembl.gramene.org/, accessed on 10 April 2025) to acquire gene information. Gene expression was quantified by counting the mapped reads for each gene. The FPKM (Fragments Per Kilobase of transcript per Million fragments mapped) metric was used to normalize and quantify transcript or gene expression. DEGs were identified using stringent criteria, namely |log2(FoldChange)| > 1.0 and adjusted *p*-value (*p*_adj) ≤ 0.05 [37]. The clusterProfiler package (version 4.2.0) in R software (https://github.com/YuLab-SMU/clusterProfiler/, accessed on 10 April 2025) was used to perform Gene Ontology (GO) and Kyoto Encyclopedia of Genes and Genomes (KEGG) enrichment analyses based on the hypergeometric test, with an adjusted *p*-value (*p*_adj) threshold of <0.05 to identify significantly enriched terms and pathways.

### 2.6. KASP Marker Development and Validation

We developed a KASP marker, FMqRrm1, based on sequence variations observed between highly resistant and susceptible inbred lines from the GWAS population on the short arm of chromosome 4 (Chr4S). This marker system comprises two allele-specific forward primers and one common reverse primer: FMqRrm1-F1 (5′-GAAGGTGACCAAGTTCATGCTGCGCTAATAAGCTAAGCTTAAGGC-3′), FMqRrm1-F2 (5′-GAAGGTCGGAGTCAACGGATTGCGCTAATAAGCTAAGCTTAAGGT-3′), and FMqRrm1-R (5′-TATAGTGCGTGATGCAGTTGAGC-3′). We used this marker for genotype analysis in the GWAS and backcross populations. Phenotypic variations among different genotypes were statistically analyzed using one-way analysis of variance (ANOVA), followed by result visualization through column graphs generated using Prism v8.0.2 software (https://www.graphpad.com/, accessed on 10 April 2025).

## 3. Results

### 3.1. Phenotypic Data Analysis for Aphid Resistance

A GWAS population of 278 inbred lines was used to evaluate phenotypic resistance to aphids across three environments. Seventeen inbred lines with high aphid resistance were identified (Table S1), laying the groundwork for future aphid resistance breeding. The analysis revealed that the frequency distribution of aphid resistance levels was relatively uniform, with a pronounced bipolar distribution. This finding shows that multiple genes with minor individual effects may control aphid resistance (Figure 1b). Correlation analysis across environments revealed correlation coefficients ranging from 0.44 to 0.88. These coefficients were notably high and statistically significant, indicating that the resistance phenotypes exhibited robust and consistent differences across environments with minimal variability. Moreover, repeatability analysis within individual environments yielded repeatability values ranging from 0.78 to 0.94, underscoring the reliability of the phenotypic measurements. The joint analysis of all environments yielded a heritability estimate of 0.77 (Table 2), suggesting that genetic factors predominantly influence aphid resistance in this population. These findings highlight the polygenic nature of aphid resistance and the stability of resistance phenotypes across varying environmental conditions.

### 3.2. GWAS

**Figure 2:**
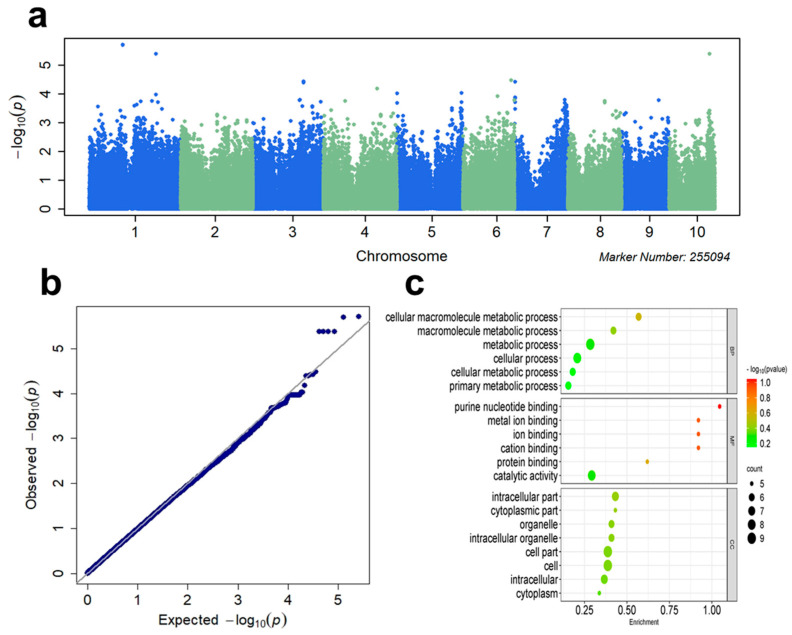
GWAS results for aphid resistance using the MLM statistical approach. (**a**) Manhattan plot displaying GWAS results. (**b**) QQ plot of GWAS results. (**c**) Bubble maps for GO enrichment analysis of significant loci. BP: Biological processes; MF: molecular function; CC: cellular component.

Using the MLM statistical approach, we performed a GWAS analysis for the combined BLUE values of the three environments. Twenty-two SNPs significantly associated with aphid resistance were identified across 10 chromosomes using a 1 × 10^−4^ standard (Figure 2a). The QQ plot indicated good control of population structure in GWAS analysis (Figure 2b). The bioinformatics annotation of the functions of the candidate genes linked to these 22 significant SNPs indicated that these genes encompass crucial components of the Bx synthesis pathway such as *Bx2*, transcription factors like *WRKY23*, plant lectin genes, and other genes pertinent to insect resistance, alongside various transcription factors (Table 3), implying a potential association with maize resistance to aphids. GO enrichment analysis was conducted to perform functional classification annotations of the candidate genes for biological processes, molecular functions, and cellular components (Figure 2c). The key molecular functions involved included cellular metabolic processes, protein binding, and metal ion binding. The primary biological processes were cellular macromolecule metabolic processes, and the cellular components were mainly enriched in intracellular and cytoplasmic components.

Compared with other diseases and pests of maize, research on the molecular mechanism of resistance to aphids is relatively scarce. The candidate genes related to aphid resistance in maize discovered through GWAS in this study involve multiple aspects. For instance, *GRMZM2G352234* encodes the pentatricopeptide repeat (PPR) superfamily protein, which regulates organelle functions and gene expression to help plants respond to environmental stress. *GRMZM2G161411* and *GRMZM2G056407* encode transcription factors WRKY23 and MYB94, respectively, and may contribute to aphid resistance by regulating the expression of downstream target genes. *GRMZM2G176133* encodes the Ribosomal protein L33 family protein. As an important component of the ribosome, this protein may enhance the aphid resistance by regulating the protein synthesis pattern related to stress responses [38]. *GRMZM2G465999* encodes the G-type lectin-like receptor kinase (LecRLK). Studies have found that LecRLKs are involved in plant disease resistance responses caused by bacteria, fungi, herbivorous insects, etc. [39]. *GRMZM2G085661* encodes the cytochrome P450 family 71B subfamily polypeptide 37 (CYP71B37). To date, the structural organization and classification of the *P450* genes have been reported in many plants such as Arabidopsis, but little is known about that in maize [40]. P450s are closely related to the biosynthesis of phytoalexins, hormone metabolism and the biosynthesis of other secondary metabolites. So, CYP71B37 maybe play a key role in the biosynthetic pathway of benzoxazinone and participate in the specific reaction step in the catalytic process of benzoxazinone synthesis.

### 3.3. RNA-Seq

**Figure 3:**
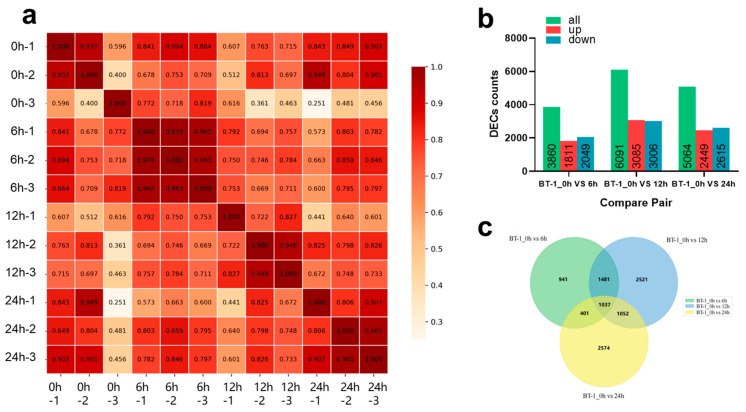
Analysis of the differentially expressed genes (DEGs) following aphid infestation. (**a**) Heatmap for correlation analysis of gene expression levels across samples. (**b**) The number of genes that show differential expression after aphid infection. (**c**) A Venn diagram of DEGs at four sampling times following aphid infestation.

We performed RNA-seq using the inbred line BT-1 at four time points (0, 6, 12, and 24 h) after aphid infestation to elucidate the genetic mechanisms of aphid resistance in maize. Table S2 summarizes the statistical analysis of the sequencing data from the 12 samples (3 biological replicates per sample). The results revealed that the number of reads per sample ranged from 42.1 to 50.16 million, with an average of 46.93 million. The average proportion of Q20 and Q30 bases was 98.20% and 94.99%, respectively, while the average GC content was around 54%. Those findings show that the sequencing data quality of all samples was excellent and appropriate for further analysis. Pearson correlation coefficients were used to analyze the repeatability of the samples (Figure 3a). A correlation coefficient above 0.8 indicated high repeatability. Samples with coefficients below this threshold were excluded from subsequent analyses to preserve the data’s integrity and quality.

Several DEGs changed significantly in response to aphid infestation (Figure 3b). At 6 h post-infestation, 1811 genes were up-regulated, while 2049 genes were down-regulated. By 12 h, the numbers had risen to 3085 up-regulated and 3006 down-regulated genes, a significant increase over the 6 h period. At 24 h post-infestation, the number of up-regulated and down-regulated genes dropped to 2449 and 2615, respectively, a decrease relative to the 12 h period. Overall, changes in gene expression levels following aphid infestation were significant, with the highest number of DEGs detected at 12 h. This suggests that aphid infestation triggers extensive physiological and biochemical responses in maize, implying the plant’s ability to adjust physiological and biochemical processes to counteract aphid infestation. Using a Venn diagram to assess the shared DEGs across three post-infestation time points (Figure 3c), 1037 genes were identified as having consistent differential expression patterns from 6 to 24 h after infestation.

**Figure 4:**
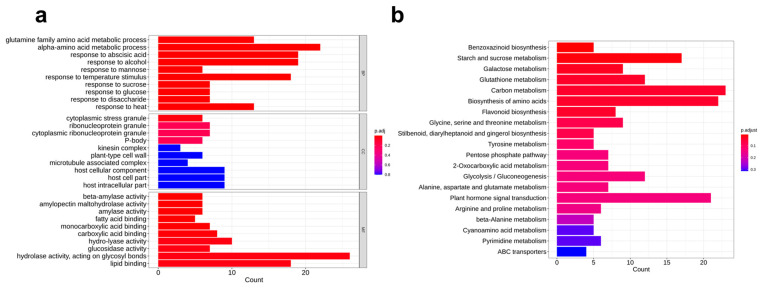
GO (**a**) and KEGG (**b**) pathway enrichment of genes that consistently respond to aphid infestation.

GO functional enrichment analysis of DEGs revealed significant enrichment in the metabolic processes of glutamine family amino acids and α-amino acids, as well as the response processes to substances such as abscisic acid, alcohol, mannose, and sucrose (Figure 4a). They also exhibited a high level of enrichment in biological processes including response to heat stress. KEGG pathway analysis was used to investigate the enrichment of metabolic pathways associated with these DEGs to clarify the physiological and biochemical processes involving them (Figure 4b). The results show that the benzoxazinoid biosynthesis pathway and the starch and sucrose metabolism pathway were among the most significantly enriched metabolic pathways (Table 4). Five benzoxazinoid biosynthesis genes with expression differences were identified, including LOC103652724 (*Zm00001eb165550*, *Bx3*), LOC103652726 (*Zm00001eb165580*, *Bx5*), LOC542117 (*Zm00001eb165610*, *Bx1*), LOC100192631 (*Zm00001eb165620*, *Bx2*) and LOC100147731 (*Zm00001eb169520*, *Bx7*). The gene *GRMZM2G085661* was jointly identified in both experiments.

In addition, the candidate gene *GRMZM2G465999* in GWAS belongs to the member of *LecRLKs*. As a pattern recognition receptor (PRR), the extracellular lectin domain of LecRLKs can recognize specific sugar molecules on the surface of pathogenic bacteria or insects, and then transmit signals into the cell through the intracellular kinase domain, activating downstream immune signaling pathways, thereby enhancing the plant’s resistance to biological stress. Due to the similarity of the recognition domain of LecRLKs to lectin in leguminous plants, it is speculated that they may be involved in carbohydrate signal recognition and transduction [39]. This coincided with the various sugar metabolisms of the KEGG pathway of DEGs following aphid infection in the transcriptome.

### 3.4. Functional Verification of the Gene Cluster on Chromosome 4S

**Figure 5:**
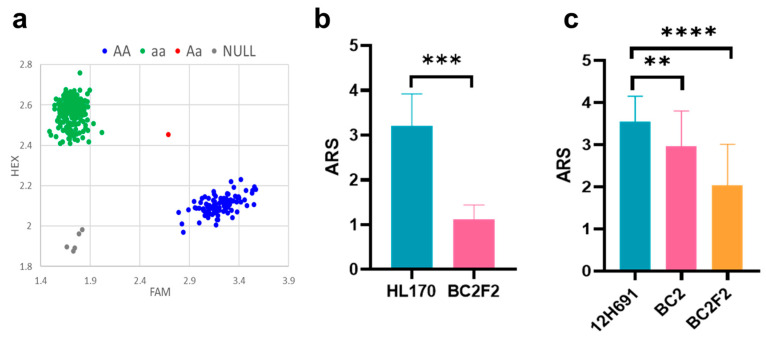
Development of aphid-resistant markers and their application in marker-assisted breeding (MAS). (**a**) Development of molecular marker, FMqRrm1, for MAS for aphid resistance. The blue dots signify the favorable allele AA associated with aphid resistance, the green dots denote the standard allele aa linked to aphid susceptibility, the red fluorescence represents the heterozygous Aa, and the gray area is the negative control. (**b**) Resistance comparison after introducing aphid-resistant chromosomal fragments into the HL170 background. (**c**) Resistance comparison after introducing aphid-resistant chromosomal fragments into the 12H691 background. Data are means ± SD from three biological replicates. ** *p* < 0.01, *** *p* < 0.001, **** *p* < 0.0001.

Integrating association analysis and RNA-seq results identified a candidate gene cluster associated with benzoxazinoid biosynthesis on the short arm of chromosome 4 (Chr4S) in maize, which exhibited a significant correlation with aphid resistance. We used KASP technology and sequence alignment of highly resistant and susceptible inbred lines to develop a molecular marker, FMqRrm1, within the 4S chromosomal region. Genotypic analysis of 458 inbred lines using the FMqRrm1 marker confirmed its effectiveness in discriminating different genotypes (Figure 5a). Our study revealed that 31.66% (145/458) of the materials possessed the aphid-resistant allele, characterized by distinct blue fluorescence signals. In contrast, 68.34% (313/458) lines exhibited susceptible alleles. Analysis of variance demonstrated that individuals carrying the resistant allele had significantly lower aphid index scores than the susceptible ones (*p* < 0.001), with an 18.5% relative gain in resistance (Table 5). These findings indicate a significant association between the FMqRrm1 marker and aphid resistance in maize.

Using the developed molecular marker FMqRrm1 and the highly resistant inbred line CNW098, we used MAS to introduce the chromosome 4 region into the susceptible inbred line HL170. Following foreground and background selection in the segregating generations (Figure 5b), the BC_2_F_2_ generation yielded a line with an 88.5% background recovery rate and a significantly reduced aphid index (from 3.21 to 1.11). Meanwhile, using the susceptible inbred line 12H691 as the recurrent parent (Figure 5c), we introgressed the resistance region from the resistant inbred line CNW098. At the BC_2_ and BC_2_F_2_ generations, the background recovery rates were 89.3% and 90.7%, respectively, while the aphid resistance score declined from 3.55 to 2.97 and 2.03, respectively. The study found that introgressing the short arm of chromosome 4 from the highly resistant CNW098 inbred line into the genetic background of susceptible materials resulted in a significant boost in aphid resistance. This finding implies that this chromosomal region contains key genes that confer aphid resistance in maize.

## 4. Discussion

Maize, a crucial economic and staple crop, is strategically essential to agricultural production. As plant density increases, aphids, one of the primary pests threatening maize growth and development, severely limit its yield [41]. Therefore, cultivating and promoting aphid-resistant maize varieties has emerged as the most cost-effective strategy for aphid control, and its implementation is dependent on identifying and utilizing highly resistant germplasm. This study successfully screened and identified 17 aphid-resistant germplasm resources, providing a significant material foundation for aphid resistant breeding.

Environmental variables influence the occurrence and population dynamics of aphids. Therefore, resistance evaluation must be conducted in regions with high aphid infestation to ensure the accuracy and reliability of the results. We selected Hainan as the primary experimental site due to its unique tropical climate conditions during the winter season, which are ideal for aphid reproduction and infestation. This ensured a stable and profuse aphid population, establishing optimal natural conditions for resistance evaluation. Using multiple years of field observations, our study systematically integrated multidimensional data, including aphid population dynamics and plant damage levels, to establish a scientific and comprehensive resistance evaluation standard. In addition to offering crucial theoretical and methodological support for aphid-resistant breeding, this study provides a solid platform for future GWAS targeted at identifying and functionally analyzing aphid-resistant genes.

Benzoxazinoids are a class of secondary metabolites widely found in gramineous crops such as maize and wheat. They are the most abundant secondary metabolites in the root exudates of these crops, providing broad-spectrum resistance against various pests and diseases [42]. Benzoxazinoids function mainly through three aspects: (1) direct toxicity and inhibitory effects on the aphids; (2) acting as signal molecules to activate maize’s defense response signaling pathways; (3) indirect defense against corn aphids by attracting their natural enemies. The most abundant benzoxazinone compound in maize is 2,4-dihydroxy-7-methoxy-1,4-benzoxazin-3-one glucoside (DIMBOA-Glc). DIMBOA-Glc and HDIMBOA-Glc are secondary metabolites present in relatively high concentrations in maize leaves, but their levels vary significantly across various maize inbred lines [43]. Studies have shown that JA promotes 2-Hydroxy-4,7-Dimethoxy-1,4-Benzoxazin-3-One Glucoside (HDMBOA-Glc) accumulation by catalyzing the conversion of DIMBOA-Glc to HDMBOA-Glc. HDMBOA-Glc subsequently degrades rapidly into DIMBOA and other active compounds that are toxic to aphids, significantly improving maize plants’ resistance to pests such as the beet armyworm, fall armyworm, and southwestern corn borer [44].

This study identified a batch of germplasm resources exhibiting significant aphid-resistance traits based on resistance studies conducted in three distinct environments. GWAS identified 22 SNP loci significantly associated with aphid resistance. We used RNA-seq technology to detect 1037 DEGs responsive to aphid infestation and further explored the related gene pathways. Through integrated GWAS and RNA-Seq analyses, it was found that the sequence variation on Chr4S was significantly associated with aphid resistance. The candidate genes corresponding to these significant SNPs are closely related to the BX synthesis pathway, which may indicate that the sequence variation of these genes may affect plant insect resistance by regulating the biosynthesis of BXs (such as DIMBOA). This study mainly focuses on the association between DNA variation and resistance. The determination of DIMBOA content in different genotypic materials in the follow-up study will provide important evidence to validate this hypothesis.

Previous research has demonstrated that at least eight genes (BX1-BX8) located on the short arm of chromosome 4 in maize regulate the DIMBOA-Glc biosynthesis pathway [7]. Integrating the results of GWAS and RNA-seq, we also detected a resistance hotspot region on the short arm of chromosome 4. With the rapid advancement of molecular biology technologies, KASP molecular marker technology has emerged as an essential tool in crop genetic research due to its high stability, cost-effectiveness, and broad applicability [45]. Based on sequence variations in the 4S chromosomal region, we successfully developed a KASP marker, FMqrm1, for genotype detection in the backcross population.

Plants counteract pests through both insect resistance and tolerance. In this study, we revealed the resistance characteristics of maize to aphids by systematically investigating the degree of coverage of aphids on maize plants. It is noteworthy that maize tolerance to aphids is equally important in the defense system, but its molecular mechanism remains to be elucidated. Recent advances have highlighted two key physiological mechanisms in plant insect tolerance: (1) increased photosynthetic activity and (2) the use of detoxification mechanisms to counteract the detrimental effects of hemipteran herbivorous insects [46]. In our study, the DEG results of the transcriptome experiment indicated that there were differential expressions of genes related to sugar/starch metabolism and other types of carbon metabolism, which may indicate that the tolerance of maize to aphids is also important, and these sugar (energy) metabolism-related genes are very likely to be involved in regulating the tolerance process of maize to aphids. However, the potential tolerance mechanisms and related gene regulatory networks also require further in-depth research.

We subsequently used MAS technology to introduce the Chr4S region of resistance inbred line CNW098 into aphid-susceptible inbred lines, significantly enhancing maize aphid resistance by 1.5–5 disease scores. Our study thus establishes crucial theoretical foundations and technical support for breeding superior aphid-resistant germplasm. This study provides vital technical support for accelerating the breeding of new maize varieties with high aphid resistance and superior comprehensive agronomic traits by introducing superior aphid resistance genes from resistant inbred lines into maize varieties with excellent agronomic traits but susceptible to aphids, thereby offering stronger varietal support for agricultural production. Future research should focus on the cloning of aphid resistance-related genes, as well as their specific functions and regulatory networks in resistance mechanisms. Our results provide a theoretical foundation for future research into maize aphid resistance-related genes and precise molecular design strategies for aphid-resistant breeding.

## Figures and Tables

**Table 1:** Classification criteria for different phenotypes.

Aphid Resistant Score	Symptom
0	No aphid
1	Aphid cover area is less than 5% of the tassel or leaf.
2	Aphid cover area is more than 5% and less than 30%, and there are very few aphids in the lower leaves.
3	Aphid cover area is more than 30% and less than 50%, and there are very few aphids in the lower leaves.
4	The aphid covers the tassel more than 50%, and there are some aphids in the lower leaves.
5	The tassels and lower leaves are covered with dense aphids.

**Table 2:** Descriptive statistics of the GWAS population for aphid resistance in three environments.

Environments	Mean ± SD	Range	σ_g_^2^	σ_ge_^2^	σ_e_^2^	*H* ^2^
ARS_E1	2.83 ± 1.76	0.00–5.00	2.74		0.32	0.94
ARS_E2	2.98 ± 1.37	0.00–5.00	1.44		0.29	0.91
ARS_E3	2.92 ± 1.58	0.00–5.00	1.61		0.85	0.78
Combine	2.91 ± 1.58	0.00–5.00	1.17	0.78	0.47	0.77

**Table 3:** List of significant SNPs detected by genome-wide association study (GWAS) and the corresponding candidate gene and its functional annotation.

Chr	Position	Gene	Function Annotation
1	219548351	*GRMZM2G127361*	Oxidoreductase zinc-binding dehydrogenase family protein
1	82154848	*GRMZM2G132690*	Heavy metal transport/detoxification superfamily protein
2	196411877	*GRMZM2G114107*	winged-helix DNA-binding transcription factor family protein
2	3387748	*GRMZM2G047448*	(PGA6, WUS, WUS1) Homeodomain-like superfamily protein
3	196434589	*GRMZM2G352234*	Pentatricopeptide repeat (PPR) superfamily protein
4	3261014	*GRMZM2G085661*	(CYP71B37) cytochrome P450 family 71 subfamily B polypeptide 37, benzoxazinone synthesis 2
4	240329225	*GRMZM2G054162*	(IBM1) Transcription factor jumonji (jmjC) domain-containing protein
5	15323527	*GRMZM2G161411*	(ATWRKY23, WRKY23) WRKY DNA-binding protein 23
5	201461600	*GRMZM2G147430*	Phosphoribulokinase/Uridine kinase family
5	147624523	*GRMZM2G026490*	NHL domain-containing protein
6	145107284	*GRMZM2G479523*	(GL22) germin-like protein subfamily 2 member 2 precursor
6	158441896	*GRMZM2G176133*	Ribosomal protein L33 family protein
6	161283910	*GRMZM2G034225*	expressed protein
7	8677546	*GRMZM2G107408*	expressed protein
7	167601337	*GRMZM2G056407*	(ATMYB94, ATMYBCP70, MYB94) myb domain protein 94
7	156116120	*GRMZM2G465999*	S-locus lectin protein kinase family protein
8	151270345	*GRMZM2G017305*	P-loop containing nucleoside triphosphate hydrolases superfamily protein
8	163281806	*GRMZM2G065893*	(RGLG2) RING domain ligase2
9	100282128	*GRMZM2G423693*	Nucleotide-diphospho-sugar transferase family protein
10	133276617	*GRMZM2G123719*	(ATSCO1, ATSCO1/CPEF-G, SCO1) Translation elongation factor EFG/EF2 protein
10	133332879	*GRMZM2G054418*	ATP binding microtubule motor family protein
10	137656350	*GRMZM2G155762*	basic helix–loop–helix (bHLH) DNA-binding superfamily protein

**Table 4:** Significant enrichment analysis of the KEGG pathway of differentially expressed genes (DEGs) following aphid infection.

Pathway ID	KEGG Pathway	Gene Number	*p*_adj
ko00402	Benzoxazinoid biosynthesis	5	5.9 × 10^−3^
ko00500	Starch and sucrose metabolism	17	1.6 × 10^−2^
ko00052	Galactose metabolism	9	3.1 × 10^−2^
ko00480	Glutathione metabolism	12	4.0 × 10^−2^
ko01200	Carbon metabolism	23	4.2 × 10^−2^

**Table 5:** The analysis of variance for the molecular marker FMqRrm1 for aphid resistance in the GWAS population.

Allele	Number	Average Disease Grade	Increased Resistance	*p*-Value
Favorite allele	145	4.75	18.50%	1.29 × 10^−5^
Normal allele	313	5.83		

## Data Availability

All datasets supporting the conclusions of this article are included in the article and Supplementary Files. Further inquiries can be directed to the corresponding author(s).

## Supplementary Materials

The following supporting information can be downloaded at: https://www.mdpi.com/article/10.3390/plants14111614/s1, Table S1. The list of high-resistance inbred lines in the GWAS population. Table S2. Data quality analysis for RNA sequencing result.
